# An interventional pilot study on obesity among low-income patients using a computer-based weight management module

**DOI:** 10.3402/jchimp.v3i1.20072

**Published:** 2013-04-17

**Authors:** Tracey Doering, Susan Harwell, Cheryl Fassler, Kesley Burr, Sara Hewitt, Christopher Trabue

**Affiliations:** Department of Medicine, University of Tennessee Health Science Center, Baptist Hospital, Nashville, TN, USA

**Keywords:** obesity, weight management, low-income

## Abstract

Primary care physicians infrequently address lifestyle modification with their obese patients, among whom those of lower economic means are disproportionately represented. To enhance patients’ access to education on lifestyle modification, a clinic-based computer kiosk was installed at our residency clinic for the purpose of healthy lifestyle education. While posttest scores improved and were maintained after completion of lifestyle modification education, body mass index (BMI) was essentially unaffected. Computer-based education without intensive counseling on lifestyle modification appears ineffective in reducing BMI amongst obese patients of lower economic means. Accountable care organization-sponsored health coaching may represent a potential means by which intensive counseling is accomplished among such patients.

Obesity has emerged as an epidemic of monumental proportion over the past decade with an estimated one-third of adults in the United States meeting the definition of obesity (BMI ≥ 30) (1). To effectively combat this growing problem, guidelines have been developed to aid primary care physicians that include counseling on lifestyle modification (2). However, there are data suggesting that relatively few primary care physicians address this issue with their obese patients (3, 4). Obesity disproportionately affects patients of lower economic means, many of whom are unable to afford structured weight loss programs, pharmacotherapy, or surgery which are the most effective means to achieve weight loss (5). To improve access to education and lifestyle modification among our obese patients of lower economic means, we implemented a computer-based weight management program and studied its efficacy.

## Methods

We conducted a quasi-experimental pilot study of 24 obese patients, examining the effect of computer-based weight management training on weight and body mass index (BMI). The study was conducted at a community-based internal medicine residency clinic affiliated to the University of Tennessee, which provides primary care to an underserved population of patients of limited economic means. Of those enrolled, 68% had either no insurance or Medicaid. Inclusion was limited to adult patients with a BMI≥30 who were at the contemplation stage or higher of behavioral change, and exclusion was based primarily on the ability to safely exercise. Patients were identified during clinic visits and enrolled by internal medicine residents, faculty, and a nurse manager. Eligible patients were referred to an onsite computer kiosk provided through a grant from the National Library of Medicine (NLM). An online pretest of five questions was administered, followed by an NLM MedlinePlus^®^ educational program on weight management, and finally a posttest of five questions. The program was designed in a similar manner to other MedlinePlus modules at a 3rd grade reading level and was accompanied by audio to accommodate illiterate patients (6). Educational material presented via the module concerned lifestyle modification involving healthy diet and exercise (Table 1) (7). Additional five question posttests were administered at 1 and 3 months. The weight of each patient was recorded at the start of the study and again at the 3-month visit. This study was approved by the institutional review board of the University of Tennessee Health Science Center.

**Table 1:** MedlinePlus^®^ weight management tutorial summary

Section 1 – Why we gain weight Basic information on energy, metabolism, and calorie content of various food types (carbohydrates, fat, and protein) Section 2 – Benefits of losing weight Summary of health benefits Improved quality of life Section 3 – How to lose weight Reducing calories consumed Increasing calories metabolized Section 4 – Tips for starting Improving eating habits with emphasis on variety, fruits and vegetables, and low fat content Increasing frequency and quality of exercise with tips on how to incorporate exercise into a daily work schedule

## Results

**Fig. 1:**
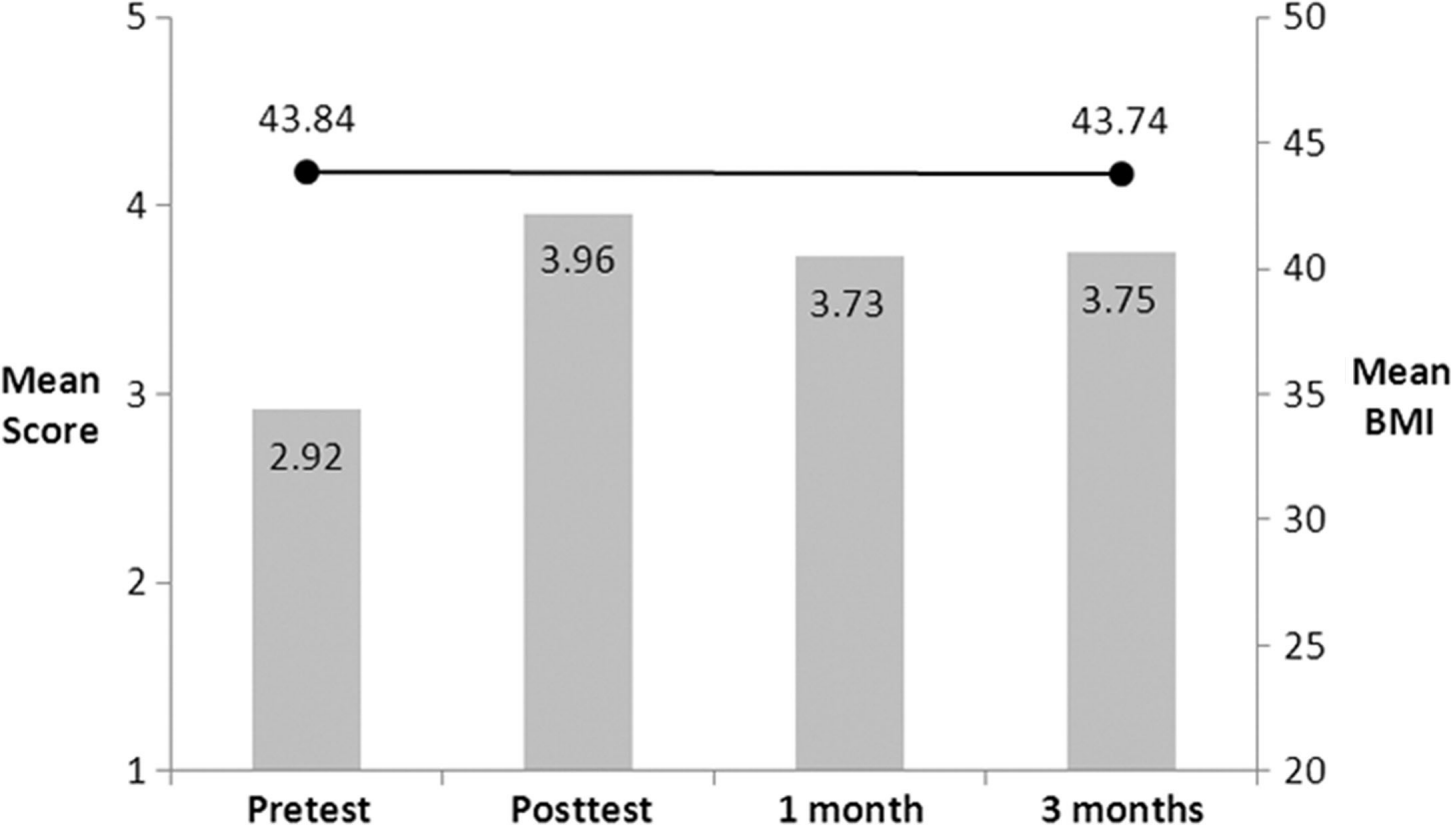
Relationship between mean BMI and test scores over a 3-month period before and after kiosk weight management training.

A total of 24 patients were enrolled with a mean BMI of 43.84 (Obesity Class III). All 24 patients completed the pretest, educational training module, and posttest; 22 and 17 patients completed 1 and 3 month posttesting, respectively. The mean pretest/immediate posttest scores improved from 2.92 to 3.96 following completion of the program, and this improvement was essentially maintained at 1 and 3 months (3.73, 3.75). A ≥5% weight loss was achieved in only 3 of 24 patients (13%). Mean and median BMI changes were negligible (−0.10, −0.44), and there was no correlation in test performance and weight loss (Fig. 1).

## Discussion

This pilot study suggests that computer-based education on lifestyle modification alone is ineffective in achieving weight loss among obese patients. While somewhat disappointing, our findings are emblematic of clinicians’ struggles with how to approach weight loss in the primary care setting. Lifestyle modification has been associated with modest weight loss and there are data that support the institution of weight loss programs or counseling in the primary care setting (8–11). Furthermore, in 2012, the US Preventive Services Task Force released a recommendation statement espousing lifestyle intervention as a grade B recommendation with an expected weight loss of 1.5–5 kg or 4% of baseline weight at 12 months (12). However, these studies incorporate intensive intervention with routine longitudinal follow-up and program maintenance (12–26 sessions annually in some) to be effective. Such programs are mainly supported by research and governmental funding and are difficult to implement and maintain in a private practice setting, where reimbursement for such services is lacking.

This small pilot study has significant limitations involving its design and scope and, therefore, caution must be used in interpreting the results. However, the goal was to favorably impact the low-income population that the clinic serves where obesity is particularly problematic and where resources to address it are lacking. While an automated process to educate patients on lifestyle modification has significant appeal, routine and intensive counseling must also accompany education to be effective (9, 10, 12).

With the advent of the accountable care organization as a part of the Affordable Care Act of 2009, health coaching has emerged as an innovative strategy to achieve long-term change with regard to lifestyle modification (13, 14). A health coach is a health care professional who works closely with patients and their physicians in implementing a plan of comprehensive lifestyle improvement. This may very well become an avenue through which meaningful and sustained weight loss is attained among obese patients and could supplant other less effective interventions.

## References

[1] Flegal KM, Carroll MD, Ogden CL, Curtin LR (2010). Prevalence and trends in obesity among US adults, 1999–2008. JAMA.

[2] Snow V, Barry P, Fitterman N, Qaseem A, Weiss K (2005). Pharmacologic and surgical management of obesity in primary care: A clinical practice guideline from the American College of Physicians. Ann Intern Med.

[3] Galuska DA, Will JC, Serdula MK, Ford ES (1999). Are health care professionals advising obese patients to lose weight?. JAMA.

[4] Vickers KS, Kircher KJ, Smith MD, Petersen LR, Rasmussen NH (2007). Health behavior counseling in primary care: Provider-reported rate and confidence. Fam Med.

[5] McLaren L (2007). Socioeconomic status and obesity. Epidemiol Rev.

[6] Teolis MG (2010). A MedlinePlus kiosk promoting health literacy. J Consum Health Internet.

[7] Patient Education Institute (2012). Weight Management. MedlinePlus Interactive Health Tutorial from the Patient Education Institute.

[8] Huerta S, Li Z, Li HC, Hu MS, Yu CA, Heber D (2004). Feasibility of a partial meal replacement plan for weight loss in low-income patients. Int J Obes Relat Metab Disord.

[9] Nanchahal K, Townsend J, Letley L, Haslam D, Wellings K, Haines A (2009). Weight-management interventions in primary care: A pilot randomised controlled trial. Br J Gen Pract.

[10] Noel PH, Wang CP, Bollinger MJ, Pugh MJ, Copeland LA, Tsevat J (2012). Intensity and duration of obesity-related counseling: Association with 5-year BMI trends among obese primary care patients. Obesity (Silver Spring).

[11] Wadden TA, Volger S, Sarwer DB, Vetter ML, Tsai AG, Berkowitz RI (2011). A two-year randomized trial of obesity treatment in primary care practice. N Engl J Med.

[12] Moyer VA (2012). Screening for and management of obesity in adults: U.S. Preventive Services Task Force recommendation statement. Ann Intern Med.

[13] Buckley PT (2011). Health coaching plays role in the ACO. Health-Leaders Media.

[14] Leahey TM, Wing RR (2012). A randomized controlled pilot study testing three types of health coaches for obesity treatment: Professional, peer, and mentor. Obesity (Silver Spring).

